# Retinotopic Mapping of Categorical and Coordinate Spatial Relation Processing in Early Visual Cortex

**DOI:** 10.1371/journal.pone.0038644

**Published:** 2012-06-19

**Authors:** Ineke J. M. van der Ham, Maarten J. A. Duijndam, Mathijs Raemaekers, Richard J. A. van Wezel, Anna Oleksiak, Albert Postma

**Affiliations:** 1 Helmholtz Institute, Experimental Psychology, Utrecht University, Utrecht, the Netherlands; 2 Department of Neurosurgery, University Medical Centre Utrecht, Utrecht, the Netherlands; 3 Helmholtz Institute and Utrecht Institute for Pharmaceutical Sciences, Utrecht University, Utrecht, the Netherlands; 4 Institute for biomedical technology and technical medicine, Biomedical Signals and Systems, University of Twente, Enschede, the Netherlands; 5 Donders Institute for Brain, Cognition and Behaviour, Biophysics, Radboud University, Nijmegen, the Netherlands; 6 Department of Neurology, University Medical Centre Utrecht, Utrecht, the Netherlands; CNRS - Université Claude Bernard Lyon 1, France

## Abstract

Spatial relations are commonly divided in two global classes. Categorical relations concern abstract relations which define areas of spatial equivalence, whereas coordinate relations are metric and concern exact distances. Categorical and coordinate relation processing are thought to rely on at least partially separate neurocognitive mechanisms, as reflected by differential lateralization patterns, in particular in the parietal cortex. In this study we address this textbook principle from a new angle. We studied retinotopic activation in early visual cortex, as a reflection of attentional distribution, in a spatial working memory task with either a categorical or a coordinate instruction. Participants were asked to memorize a dot position, with regard to a central cross, and to indicate whether a subsequent dot position matched the first dot position, either categorically (opposite quadrant of the cross) or coordinately (same distance to the centre of the cross). BOLD responses across the retinotopic maps of V1, V2, and V3 indicate that the spatial distribution of cortical activity was different for categorical and coordinate instructions throughout the retention interval; a more local focus was found during categorical processing, whereas focus was more global for coordinate processing. This effect was strongest for V3, approached significance in V2 and was absent in V1. Furthermore, during stimulus presentation the two instructions led to different levels of activation in V3 during stimulus encoding; a stronger increase in activity was found for categorical processing. Together this is the first demonstration that instructions for specific types of spatial relations may yield distinct attentional patterns which are already reflected in activity early in the visual cortex.

## Introduction

The ability to process spatial relations between objects in our environment is crucial to successfully execute behaviours like navigation, visual search, and object location memory. Spatial relations can be divided into two types; categorical and coordinate relations. Categorical spatial relations concern the abstract relations between objects and object parts, capturing relative spatial properties such as containment, direction, connectedness (e.g. the chair is *next to* the table). In contrast, coordinate spatial relations involve precise and metric properties, making use of exact distances, such as ‘the car is *two meters away from* the house’. Importantly, these two types of spatial relations are thought to be processed by two at least partially different subsystems. This dissociation is shown by lateralization patterns in the brain [1]. The left hemisphere is typically better in processing categorical information, whereas the right hemisphere commonly shows an advantage in processing coordinate information (see [2] for a review). This dissociation as exposed by opposing hemispheric biases, has been shown convincingly in behavioural experiments (e.g. [3]–[6]), but also by means of neuroimaging and TMS measurements (e.g. [7]–[9]).

A range of different types of experimental and stimulus designs have been applied to study the dissociation between categorical and coordinate processing. The initial experimental designs used to test spatial relation processing mainly concerned perceptual, or single stimulus, tasks (e.g. [10]). Yet, some problems emerged in these types of design; categorical decisions were too easy or practice effects were found when a large number of coordinate trials was presented (see e.g. [7]). Accordingly, in recent years more studies have applied a working memory design, involving two sequentially presented stimuli (see e.g. [11], [6]), which have to be compared following a categorical or a coordinate instruction. The results of these studies show that the same lateralization patterns are found for working memory and perceptual tasks alike, and that the working memory tasks provide a better alternative by avoiding some of the problems found for perceptual tasks.

It could well be that lateralization differences are not the only way the dissociation between categorical and coordinate processing is expressed. We suggest that the particular spatial relation instructions in these tasks affect to which location spatial attention is directed, which in turn modulates the specific contents of working memory. It has been shown that spatial working memory and spatial attention are closely linked as some have argued that such selective spatial attention functions as a rehearsal mechanism for spatial working memory (e.g. [12]–[15]). Furthermore, Munneke, Heslenfeld, and Theeuwes [16] pointed out that whereas these two constructs might be conceptually different, they were in fact very similar with regard to the underlying neural mechanisms. This claim was supported by their finding that retaining spatial information in working memory is related to retinotopic BOLD responses in early visual cortex (V1, V2, V3), congruent with findings on spatial attention (e.g. [17]). Thus, such retinotopic patterns in these areas of the visual cortex can be considered reflections of the distribution of attention, which in turn indicates working memory rehearsal.

Distribution of attention and spatial relation processing have been studied in a number of recent experiments. It has been suggested that there is a link between categorical and coordinate processing and local and global processing, respectively (e.g. [18]). The idea of such a link is strengthened by the fact that a left hemisphere advantage is commonly found for both local and categorical processing, and a right hemisphere advantage for both global and coordinate processing (for a review see [19]). Furthermore, recent behavioural findings have shown that a smaller attention window is used for categorical relation processing, as compared to coordinate relation processing (e.g. [20]–[22]). Importantly, these findings are restricted to experimental designs in which the size of the attention window was actively manipulated. An intriguing question that arises from this is whether instructions to focus on different aspects of spatial information result in a spontaneous adjustment of attentional window. Based on the foregoing, the distribution of attention during a working memory task can be determined by studying how cortical activity in early visual cortex is modulated during the task. Therefore, the main goal of the current study was to measure the retinotopic BOLD responses to categorical and coordinate spatial relation task instructions across early visual cortical areas V1, V2, and V3, in a working memory task.

The working memory task used here comprises the sequential presentation of two stimuli that participants should compare based on either the categorical or coordinate characteristics. In such measurements, activation during the interval between the two stimuli is of particular interest. The processing that takes place during this interval has been studied before in relation to strategy use, or in other words in what modality relevant information is rehearsed in memory in order to provide a correct answer. It has been shown that participants report to use more spatial strategies for coordinate processing, whereas more verbal strategies are typically used to memorize categorical information [6]. More recent work however, suggests that the difference between categorical and coordinate spatial relation processing is of a visuospatial rather than a verbal nature [23], [24]. These findings illustrate that the processing of both categorical and coordinate information is spatial in nature, so the differences between the two are based on differences in how spatial features of visual information are processed and represented. Combined with the foregoing, this stresses the importance of studying the difference between categorical and coordinate processing within the spatial domain, more specifically by studying the spatial distribution of attention.

Our experimental approach is similar to the one of Munneke et al. [16], who observed a stronger BOLD response for target locations compared to non-target locations, in the absence of visual stimuli, during the retention interval between the sequential presentation of the two stimuli. This was interpreted as an attentional rehearsal mechanism to keep spatial information available in working memory. As such, the question here is whether such attentional rehearsal mechanisms function in the same way for categorical information as for coordinate information or not. If there is a strong and fundamental difference in the way the two types of information are processed, a difference in the accompanying rehearsal mechanism is expected as well. In line with the behavioural findings concerning the size of attentional windows, it is expected that activity in early visual cortex is focused on a smaller area for categorical processing, and a larger area for coordinate processing. In other words, attention is expected to be focused more locally for categorical processing and more globally for coordinate processing, as reflected by differential activation levels in early visual cortex. In contrast, if categorical and coordinate relation processing do not differ qualitatively and do not rely on distinctive processing mechanisms, they should not differ in the way this low level rehearsal mechanism is used either. In this case, no difference should be found in the way cortical activity is distributed during the retention interval. Furthermore, as the retinotopic mapping approach covers V1, V2, and V3, its outcome can also address the issue of how early in the visual system these potential differences arise, which will provide more detailed information about these voluntary, top-down attentional processes.

## Methods

### Participants

Ten healthy subjects (three female), with a mean age of 23.8 (SD = 4.4) participated in the experiment. All participants gave informed consent for participation, approved by the local ethics committee of the University Medical Centre Utrecht, in accordance with the Declaration of Helsinki. Right handedness was ensured for all participants by means of the Edinburgh Handedness Inventory [25], with a mean score of 84.6 (SD = 23.7), on a scale of −100 (extremely left handed) to 100 (extremely right handed).

### Scanning Protocol and Apparatus

Scanning was performed on a Philips Achieva 3T scanner (Philips Medical Systems, Best, the Netherlands) with a Quasar Dual gradient set. For functional images in the retinotopic mapping procedure, a navigated 3D-PRESTO pulse sequence was used [26], [27]. The acquisition parameters were as follows: TR = 30 ms (time between two subsequent RF pulses; for PRESTO the TR is not equal to the acquisition time of a single volume); effective TE (time when the central lines of k space are acquired) = 43.87 ms; FOV (anterior-posterior, inferior-superior, right-left) = 65×200×160 mm^3^; flip angle = 10°; matrix: 26×80×64 slices; voxel size 2.5 mm isotropic; eight channel head coil; SENSE factors = 2.0 (left-right) and 1.8 (anterior-posterior). A new volume was acquired every 540 ms, and encompassed the posterior 65 mm of the brain. For the functional images of the experimental task an EPI scan sequence was used, with the following parameters; TR = 1500 ms; effective TE = 30 ms; FOV (anterior-posterior, inferior-superior, right-left) = 60×160×160; flip angle = 70°; matrix: 24×64×64 slices; voxel size 2.5 mm isotropic. EPI images encompassed the posterior part of the brain. A T1-weighted structural image of the whole brain (voxel resolution = 0.875×0.875×1.00 mm^3^) was acquired at the end of the functional series.

### Retinotopic Mapping Stimuli

**Figure 1:**
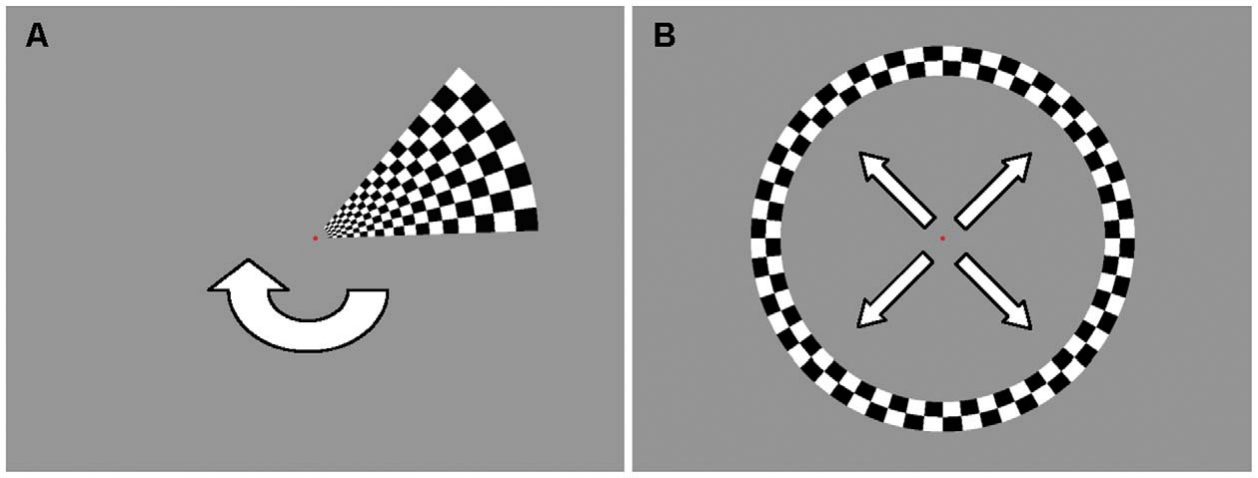
Schematics of the stimuli used for mapping. A) A clockwise rotating wedge for polar angle mapping. B) An expanding circle for eccentricity mapping. Arrows are for illustration purposes only.

For task presentation, we used a desktop PC, a projection screen, and a video-projector system. The stimuli used for retinotopic mapping were programmed in C++ software (Bjarne Stroustrup, 1983, Bell Laboratories, USA) (see Figure 1). The start of each series of stimuli was triggered by the scanner. During all retinotopic mapping stimuli, a red central fixation dot (with a radius of 0.08° visual angle) was presented, surrounded by a circular aperture (radius of 0.4° visual angle). Subjects were requested to maintain their gaze on the fixation dot regardless of the presented stimuli. The average luminance of the entire screen (42.2 cd/m^2^) was constant during all stimuli.

For eccentricity mapping, an expanding ring was used with a maximum eccentricity of 7.5° visual angle (Fig. 1A). After the ring was fully expanded, it returned to its minimum eccentricity (0.4° visual angle). The width of the ring was 1/5th of the maximum stimulus radius. There was one series (800 images) with eight cycles of 54,000 ms (100 images). Furthermore, there was a blank period with only the fixation dot during the first and last 27,000 ms (50 images) of the entire session. For polar angle mapping, a rotating wedge (45° circular angle) was used that extended to a maximum eccentricity of 7.5° visual angle (Fig. 1B). There was one series (800 images) with eight full clockwise rotations that lasted 54,000 ms (100 images) each. The screen was blank during the first and last 27,000 ms (50 images) of the series, except for the central fixation dot. Both the rotating wedge and the expanding ring contained a checkerboard pattern with white and black squares that reversed contrast every 125 ms.

### Procedure and Experimental Stimuli

**Figure 2:**
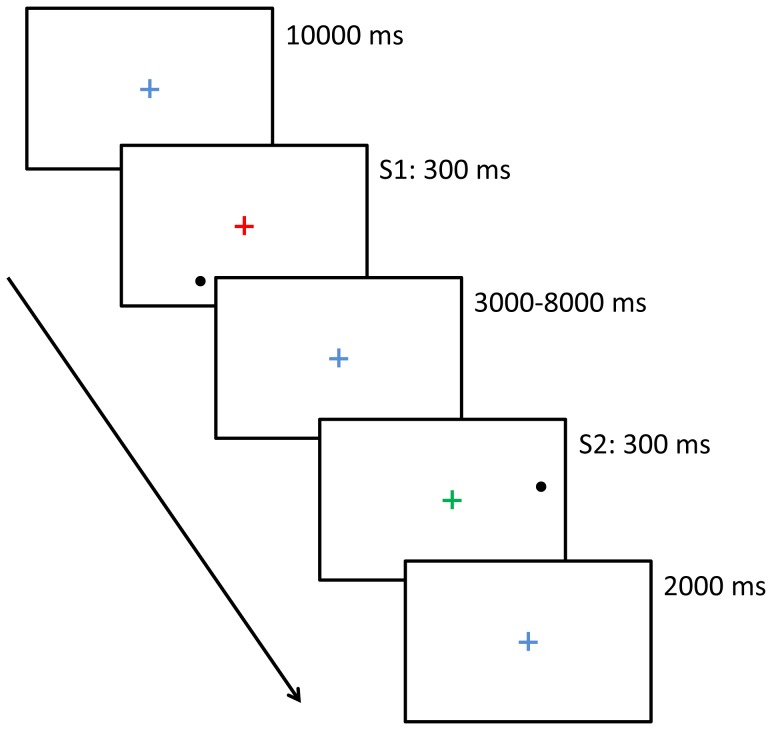
All elements of the trialsequence: an interval (10000 ms), first stimulus presentation (300 ms), jittered interval (3000–8000 ms), second stimulus presentation (300 ms), and a fixation cross during which a response could be given (2000 ms).

The task used to assess categorical and coordinate spatial relation processing was the cross dot task [6], [9], [23]. This task entailed the sequential presentation of two cross dot stimuli that participants needed to compare based on either their categorical or coordinate spatial properties. In Figure 2, a schematic depiction of the trial sequence is provided. First, a fixation cross was presented (1000 ms), followed by the first cross dot stimulus (300 ms), a second fixation cross presented during a jittered interval (3000–8000 ms), the second cross dot stimulus (300 ms), and a fixation cross presentation during which a response should be given (2000 ms). All fixation crosses and cross dot stimuli were presented centrally. The interval was jittered in order to de-correlate BOLD responses from the different neural events in the experiment.

**Figure 3:**
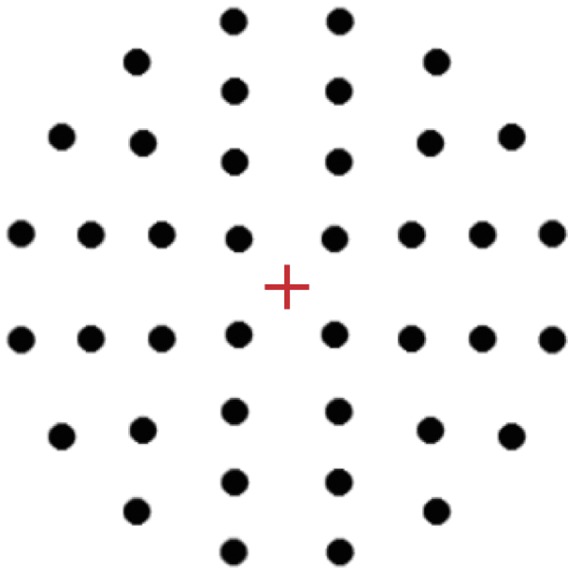
The cross dot stimulus with all possible dot positions. The central cross was red in all first stimuli (S1) and green in all second stimuli (S2). Note that only one of these dots was present in a single stimulus.

The cross dot stimuli were created with Presentation software (Neurobehavioral systems, Albany, CA). A single stimulus consisted of a cross (1.23 * 1.23° visual angle) and one dot (radius 0.92° visual angle), in Figure 3 all forty possible dot positions are presented. A same size blue cross was used as a fixation cross, before and after stimulus presentation. The cross in the first of the two stimuli within a trials (S1) was coloured red, the cross in the second stimulus (S2) was coloured green, to prevent potential mistakes in the order of stimuli within a single trial, as subjects could accidentally perceive an S2 stimulus as the S1 of the subsequent trial. The dots were black for both S1 and S2 stimuli. The dot positions were placed at four different radial distances from the centre of the cross (1.9, 3.8, 5.6, and 7.5° visual angle) and they were equally distributed over the four quadrants of the cross. Each possible position was presented twice as S1 and twice as S2; once in a match (target) trial and once in a non-match (non-target) trial.

The instruction for the categorical task was to compare the quadrants of the cross that the dots were in for two sequentially presented cross dot stimuli. A match response would be given if the dot in the second stimulus appeared in the opposite quadrant of the dot in the first stimulus, e.g. in the bottom left quadrant in the first stimulus, and the top right in the second stimulus. A non-match response should be given when the dot appeared in one of the other three quadrants. This differed from prior versions of the cross dot task (e.g. [6]) where stimuli would match if the dot was presented in the same quadrant. This change was made to be able to discriminate possible residual activation from the visual input from activation reflecting attentional processes during the task. As the matching quadrant was not the same as the quadrant present in the first stimulus, they could easily be separated in the retinotopic mapping process. The coordinate instruction was to compare the two dot positions based on the distance between the dot and the centre of the cross, regardless of quadrant. A match response should be given when the distances were the same in the two stimuli, and a non-match response when the distances were different. Thus, the matching region was a ring around the cross, with the distance between the dot and the cross as its radius. The non-matching region was the area not covered by this ring, with smaller and larger radiuses. There were four possible distances, but subjects were told that the dots could appear at any location around the cross. The selection of S1 and S2 stimulus combinations was performed at random: for the categorical instruction, the coordinate features were not taken into account, for the coordinate instruction, the categorical features could be disregarded. Subjects were informed verbally and with written instructions on the screen before the start of each scanning session about whether they had to perform the task according to the categorical or the coordinate instructions. The same type of instructions applied to the entire session, to avoid possible task switching effects.

Prior to scanning participants were trained on the cross dot task until they fully understood the instructions. After training in a regular table top set-up, participants were placed in the scanner for the actual experiment. First, both the eccentricity and polar retinotopic mapping tasks were performed. Subjects were instructed to keep their gaze at the fixation dot throughout stimulus presentation. Second, the categorical and coordinate versions of the cross dot task were shown, in counterbalanced order between subjects. In each task 80 trials were shown; 40 match trials, 30 non-match trials, and 10 catch trials. Catch trials were used to control for random noise and did not require a response from the participant. Responses were given by pressing either the left or right button of a response box with the thumb of the dominant right hand.

### Imaging Data Analysis

All preprocessing steps were done using SPM5 (http://www.fil.ion.ucl.ac.uk/spm/). After realignment, the functional images were coregistered and resliced to the FA27 volume, using the first functional volume as a source. The T1 structural image was also coregistered to the FA27-image, thereby providing spatial alignment between the structural image and the functional volumes. Low frequency noise in the fMRI data was modelled and removed from the data using a general linear model (GLM) and a design matrix containing the mean of each image and eight cosine functions forming a high-pass filter with a cutoff at 8.2×10−1 Hz.

For polar angle and eccentricity mapping, a vector was created that represented cyclic activation during presentation of wedges and rings (7,200 ms activation every 54,000 ms) and was convolved with a hemodynamic response function [28]. The cross correlation between the fMRI data and this vector was calculated for every voxel and for 100 lags (0–99; i.e. every image within a cycle) and the peak cross correlation determined the receptive field location of the voxel in polar angle and eccentricity.

The polar angle measures of voxels were used to construct the matching and mismatching regions for the categorical task. The polar angle measures were interpolated to four steps (0–90°, 90–180°, 180–270°, 270–360°), corresponding to the four quadrants of the cross in the stimuli. The eccentricity measures of voxels were used to construct the matching and mismatching regions for the coordinate task, based on the four possible radiuses of dot position (1.875, 3.75, 5.625, and 7.5° visual angle from the centre of the cross). The voxels that were significantly activated during the retinotopic mapping (p<.05; Bonferroni-corrected) formed a visual field representation that consisted of four segments, which were further subdivided in V1, V2, and V3. For each subject the average BOLD response was calculated in these segments, during S1 presentation, throughout the interval between S1 and S2, and during S1 presentation.

### Segmentation of Retinotopic Areas

The T1 image was corrected for intensity inhomogeneities using the segmentation utility in SPM5 [29]. The bias-corrected T1 images were then imported in the Computerized Anatomical Reconstruction and Editing Tool Kit (CARET) [30]. T1 images were resliced to 1 mm isotropic resolution, manually placed in Talairach orientation, and subdivided in left and right hemisphere. All subsequent steps were done per hemisphere. The intensity of the grey/white matter border was determined, followed by automatic extraction of the cortex. A white matter segment was generated and was automatically corrected for topological errors. Remaining topological errors were removed manually. A surface reconstruction was generated and inflated. Several cuts were applied on the inflated surface, amongst others along the calcarine fissure and the medial wall. The surface was flattened and geometric distortions were reduced. Results of the polar angle mapping were mapped to the anatomical surface by giving each node of the surface the value of the voxel it was located in. Retinotopic areas V1, V2, and V3 were manually segmented by drawing borders along the reversals in the change of the polar angle representation. The resulting flat segments were converted back to volumetric format and used as ROIs in further analysis.

### Statistical Analysis

Behavioural performance was analysed by means of paired sample t-tests on both the accuracy and response times. Performance was compared between the categorical and coordinate task instructions.

For the imaging data, the distribution of attention was operationalized as the distribution of cortical activity over the matching and non-matching regions of the stimuli, based on the instruction and the features of the first stimulus. For the main analyses, the retention interval between the two stimuli is used to analyse the distribution of cortical activity. In addition, the responses related to the presentation of both the first and second stimuli were analysed, to further examine the mechanisms underlying encoding and retrieval for both instructions.

Mean time courses for each ROI were extracted from the data by averaging the signal of all voxels within an ROI for each scan. The time courses of the four segments of each visual cortical area were concatenated in a single time-course with a length of four times the duration of the experimental session. A design matrix was generated with 4 factors that modelled BOLD signal changes across the entire length of 4 time-series, thereby simultaneously estimating a model for all 4 segments. The use of concatenated time series with a concatenated design matrix allowed us to test a specific model across the entire visual field by taking into account where in the visual field S1, S2, matching, and mismatching activation is expected. Furthermore, by performing the analysis on concatenated time courses, we avoided intercorrelations between factors of the design matrix (such as between stimulus presentations and delay periods), that would exist when the time courses of the four segments were analysed sequentially. There were two separate factors that represented neural activation between the first and second stimulus and consisted of boxcar functions convolved with a hemodynamic response function. This was either delay activity in the matching location or delay activity in the mismatching location. As the time courses were concatenated, the segment of the visual field where either matching or non-matching activation was expected, determined the location in the time course where the activation was modelled. The two other factors represented activity in relation to S1, or S2 which were single events convolved with a hemodynamic response function. Similarly to the modelling of the location in time in the model was also dependent on the segment where S1 and S2 activation was expected.

An additional factor was added to the model representing activation related to S1 and S2 stimuli, parametrically modulated with the visual eccentricity. This factor could explain variability in BOLD responses as a result of potential differences in the amplitude between BOLD responses to S1 and S2 of the cortical magnification factor. In the second level analysis both activation during the interval and during stimulus presentation were addressed. The beta values measured during the delay period were calculated separately for both instructions (categorical, coordinate) and for the two types of regions (matching the first stimulus according to the instruction, not matching the first stimulus according to the instruction). A general linear model including ROI, instruction, and region as within subject factors was used to analyse in what retinotopic region the activity level was higher during the retention interval between the first and second stimulus.

Furthermore, beta values directly resulting from stimulus presentation were also analysed. A general linear model including instruction (categorical, coordinate) and stimulus (S1, S2 matching region, S2 mismatching region) as within subject factors was conducted for each of the three ROIs (V1, V2, V3). This allowed for the examination of retinotopic responses related to stimulus presentation, split up by the different instructions.

Given the different spatial nature of the instructions, quadrants versus radial distance, the determined ROIs differed for the two instructions. To exclude the possibility that the differences in the ROI definition have a confounding effect on potential differences between the two instruction conditions, an additional analysis was included. For each stimulus the ‘contrast activation’ was determined, which reflects the difference in activity between the areas where the stimulus is presented versus where it is not presented. For S1 and S2 stimuli during both conditions, contrast activation was determined based on the categorical properties (quadrants) as well as coordinate properties (radial distance). If the method to determine the ROIs is equally reliable, then no difference between contrast activation should be found.

## Results

### Behavioural Results

In Table 1, the mean accuracy and response times are given for both conditions. Importantly, performance as measured by accuracy was clearly above chance level (50%) in both cases. Participants were more accurate, t(9) = 8.75, p<.001, and faster, t(9) = 2.28, p = .049, for the categorical condition, compared to the coordinate condition.

**Table 1:** Mean accuracy (Acc) and response times (RT) for both the categorical and coordinate condition.

Condition	Acc (in %)	RT (in ms)
Categorical	96.86 (3.14)	903.56 (176.36)
Coordinate	81.00 (5.64)	1081.40 (227.19)

Standard deviation in parentheses.

### Imaging Results

**Figure 4:**
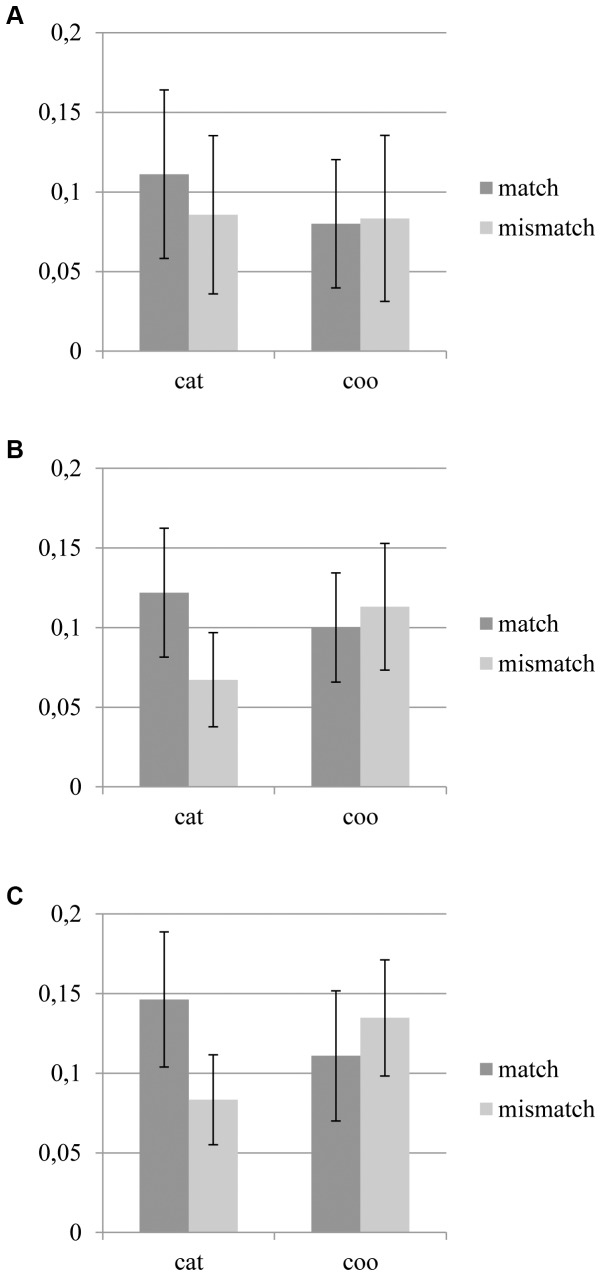
Mean regression coefficients of the retention interval between the first and second stimulus for A) V1, B) V2, and C) V3. Means are presented for both the categorical (cat) and coordinate instruction (coo) as well as the regions that would match and mismatch in comparison to the first stimulus. Error bars represent the standard error of the mean (SEM).

Hemodynamic brain responses evoked during the interval between the presentation of the first and second stimulus were determined. In Figure 4A, 4B, and 4C the mean regression coefficients for both the categorical and the coordinate conditions are depicted, for all three visual areas, and the matching and non-matching regions. A GLM including ROI, instruction, and region as within subject factors was carried out. Results from the GLM showed a significant three-way interaction of all three factors, F(2,9) = 4.39, p<.05, whereas none of the other main and interaction effects reached significance (p>.10). This three-way interaction was followed up by GLMs including both instruction and region as within subject factors, for each ROI. For V1 no significant effects were found. For V2, the significance of the interaction of instruction and region was at trend level, F(1,9) = 3.59, p<.10. Figure 4B indicates that for the categorical instruction the mean regression coefficient was higher for the matching region (if tested directly p<.05), compared to the mismatching region, whereas no such difference is visible for the coordinate instruction. For V3, the interaction of instruction and region was significant, F(1,9) = 6.06, p<.05. Post hoc tests showed that the difference between matching and mismatching regions was significant for the categorical instruction: the mean regression coefficient was higher for matching region, compared to the mismatching region (p<.05). For the coordinate instruction no significant difference between the matching and non-matching regions was found (p>.10).

**Figure 5:**
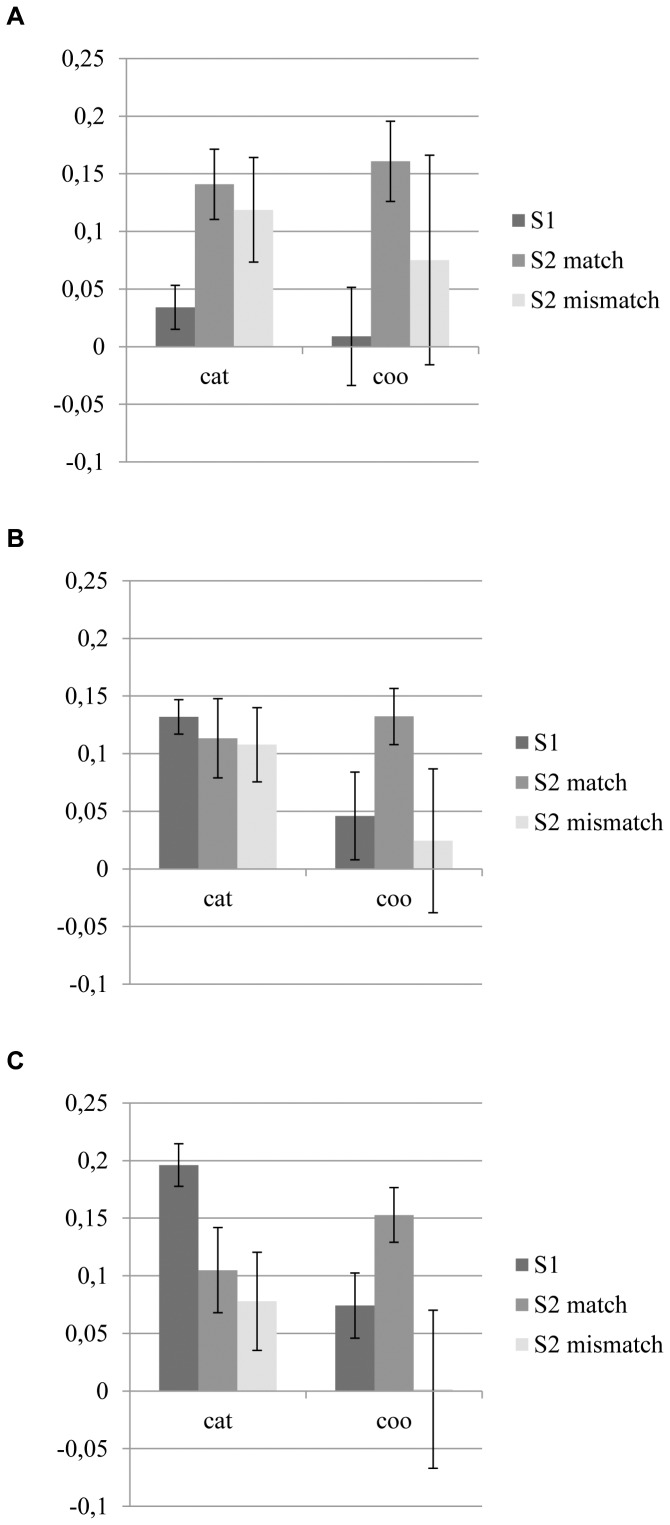
Mean regression coefficients related to all three stimulus types for A) V1, B) V2, and C) V3. Means are presented for both the categorical (cat) and coordinate instruction (coo) as well as the first stimulus (S1), the matching region of the second stimulus (S2 match), and the mismatching region of the second stimulus (S2 mismatch). Error bars represent the standard error of the mean (SEM).

To look further into the direct effect of stimulus presentation, the GLM including instruction (categorical, coordinate) and stimulus (S1, S2 matching region, S2 mismatching region) was performed for all three ROIs. In Figure 5A, 5B, and 5C the mean Beta values for both the categorical and the coordinate conditions are depicted for each stimulus type and for all three visual areas. In the regression coefficients of V1 no significant effects were found. For V2 a significant main effect of instruction, F(1,9) = 6.92, p<.05, was found; a higher regression coefficient was found for the categorical instruction compared to the coordinate instruction. For V3 a trend for instruction, F(1,9) = 3.68, p = .087, and stimulus, F(2,8) = 4.26, p = .061, was found. The regression coefficient is slightly higher for the categorical instruction, compared to the coordinate instruction. No specific differences between the three stimulus types were found.

The reliability of the ROI selection was checked by assessing the contrast activation for both the categorical and the coordinate instruction, based on both the categorical (quadrants) and coordinate (radial distance) properties of the stimuli, leading to four separate mean contrast activation levels: categorical instruction – quadrant (M = .08, SD = .03), categorical instruction – radial distance (M = .08, SD = .02), coordinate instruction – quadrant (M = .09, SD = .01), coordinate instruction – radial distance (M = .08, SD = .03). An ANOVA was used to compare all four contrast activation levels, it showed there were no differences between the levels (p>.10).

## Discussion

The goal of the present study was to study the distribution of early visual cortex activation during categorical and coordinate spatial relation processing. Specifically, we were interested in exploring whether activation patterns differ retinotopically between both types of spatial relation processing, revealing patterns of attentional focus during processing. If such a difference is found, this would be one of the first demonstrations that the two types do not only differ in lateralization, but also in the way attention is distributed over stimuli. This can be considered as complementary evidence that categorical and coordinate processing relies on distinctly different processing mechanisms. As lateralization differences have been described convincingly in numerous studies (see e.g. [31]) and are not within the main focus of this experiment, they were not included in the current analyses.

Our results indicate that retention of categorical and coordinate spatial information is handled differently in the visual cortex. In area V3 a significant interaction of instruction and region was found; when a categorical instruction is given, a higher level of activity was found in the matching region, compared to the non-matching region. For the coordinate task activity seems evenly spread over the matching and mismatching regions. This indicates that for the categorical task, subjects focus on the region that is related to a match response, whereas for the coordinate task participants differ in their focus on the matching and non-matching region. Importantly, the categorical instruction was that two stimuli would match if the dots were presented in opposite quadrants. Therefore it can be excluded that the heightened level of activity during the interval simply represents the visual rehearsal of the first stimulus. This also indicates that the process of determining the matching, opposite quadrant directly follows the first stimulus presentation, as this finding is based on the mean activity of the entire retention interval.

Interestingly, the differential effects between both task types are not found for all three ROIs combined, but they appear to grow stronger for the higher visual areas. They were clearly not present for V1, emerged at trend level in area V2, and were clearly significant in V3. This suggests a top-down influence on the direction of attention during task performance in the form of an attentional bias, facilitating responses to the attended region (e.g. [32], [33]).

The effect of a specific focus for categorical processing and the absence of such a specific focus for coordinate processing is in line with the local versus global processing distinction. The current finding can be framed as a more local focus during categorical processing and a more global focus during coordinate processing, in line with recent behavioural findings (e.g. [20]–[22]). The current study provides a valuable contribution to this line of research as attentional focus is monitored without interference, instead of manipulating the size of the attentional window as done in the previous behavioural studies. This recent line of studies should be taken into consideration when studying basic dichotomies in perception, as there might be a more basic processing mechanism underlying them, explaining higher level differences.

Importantly, the foregoing may contribute to the discussion about how lateralization patterns of spatial relation processing have emerged during evolution. Originally, Kosslyn [1] suggested that pre-existing qualities of the two hemispheres concerning language and navigation led to this differential specialization of the hemispheres with regard to spatial information. In contrast, later accounts point towards differences in receptive field sizes between the two hemispheres as the cause for lateralization (e.g. [34]). However, the present results suggest that lateralized attentional biases may play a critical role rather than hard-wired anatomical differences (e.g., [35], [29]).

In a secondary analysis the retinotopic responses to the two stimuli in the trials (S1 and S2) were addressed. The only significant effect was that of instruction in V2, where the categorical instruction was associated with higher overall activation, compared to the coordinate instruction. This effect may appear contradictory given the difficulty difference indicated by the behavioural measures, however, it may be due to the framing of the instruction. Additional processing may be needed to determine the opposite, matching quadrant, instead of merely perceived the quadrant in the first stimulus. Apart from this effect, instruction does not dissociate in activation related to the different stimulus types. This indicates that in the current set up, the differences between categorical and coordinate processing are primarily linked to the retention interval between the first and second stimulus.

The above mentioned effect brings forward the matter of task difficulty, as is very commonly found, the coordinate task was clearly more difficult than the categorical task. Some have argued that dissociations found could be attributable to difficulty instead of qualitative differences (e.g. [36]). However, in other findings (e.g. [9], [37], [38]) this difficulty in itself cannot be considered the main determinant in the differences observed. Furthermore, it should be mentioned that fixation was not monitored during the experiment. However, in a previous version of the same task, ERP data clearly indicated central fixation throughout the experiment.

Importantly, the effects reported here are unlikely to be related to the selection of ROIs. Due to the nature of the task design, the categorical and coordinate instructions lead to the selection of different ROIs: quadrants and radial distances. However, the analyses of contrast activity showed that the ROI definitions discriminated different visual field locations equally well.

In conclusion, categorical and coordinate processing mechanisms appear to differ with regard to how attention is distributed over matching and mismatching regions, in the absence of visual stimuli. Memorizing categorical information is related to specifically attending matching regions, as reflected by modulation of cortical activity, while no clear pattern in the distribution of activity in early visual cortex is found in memorizing coordinate information. This suggests a close link between a local focus and categorical processing on the one hand and a global focus and coordinate processing on the other. Furthermore, it allows for the intriguing possibility that lateralization of such cognitive mechanisms may derive from a single underlying distinction in basic attentional mechanisms.
